# Iberian pig adaptation to acorn consumption: II. Net portal appearance of amino acids

**DOI:** 10.7717/peerj.6137

**Published:** 2018-12-18

**Authors:** Manuel Lachica, Jose Miguel Rodríguez-López, Lucrecia González-Valero, Ignacio Fernández-Fígares

**Affiliations:** 1 Department of Physiology and Biochemistry of Animal Nutrition, Estación Experimental del Zaidín, Consejo Superior de Investigaciones Científicas, Granada, Spain; 2 Départment Sciences Agronomiques et Animales, Institut Polytechnique LaSalle Beauvais, Beauvais, France

**Keywords:** Pig, Net portal appearance, Portal-drained viscera, Acorn, Amino acid

## Abstract

In Iberian pig outdoor production, pigs are fed equilibrated diets until the final fattening period when grazing pigs consume mainly acorns from oak trees. Acorns are rich in energy but poor in crude protein where lysine is the first limiting amino acid (AA). Net portal appearance (NPA) is very useful to ascertain AA available for liver and peripheral tissues. The aim of this study was to determine NPA of AA in Iberian gilts fed with acorns and to ascertain if there was an effect of acorn feeding over time. Two sampling periods were carried out (after one day and after one week of acorn feeding) with six gilts (34 kg average BW) set up with three catheters: in carotid artery and portal vein for blood sampling, and ileal vein for a marker infusion to measure portal plasma flow (PPF). Pigs were fed at 2.5 × ME for maintenance a standard diet in two meals, at 09:00 (0.25) and 15:00 h (the remaining 0.75). The day previous to first sampling, pig diet was replaced by 2.4 kg of acorn. A serial blood collection was done at −5 min, 0.5, 1, 1.5, 2, 2.5, 3, 3.5, 4, 5 and 6 h after feeding 0.25 of total daily acorn ration. Following identical protocol, one week later the second sampling was done. NPA of sum of essential AA (EAA) was poor. Although increased NPA of histidine (*P* < 0.001), leucine, phenylalanine and valine (0.05 < *P* < 0.08) was found after one week of acorn consumption, the sum of EAA did not change. Furthermore, fractional absorption (NPA/AA intake) of EAA, non-essential AA (NEAA) and total AA was 97, 44 and 49% lower, respectively, at the beginning of eating acorn than a week later. Supplementation, with some of the EAA and NEAA to Iberian pigs during the grazing period would be beneficial to overcome the increased portal-drained viscera (PDV) utilization of AA observed in the present study.

## Introduction

There is a growing interest in semi-extensive and extensive production systems where an efficient use of feed resources constitutes a key factor for sustainability and animal welfare. Iberian pig (*Sus mediterraneus*) is a rustic breed that thrives in the environmental conditions of the Mediterranean forest in the Southwest of Portugal and Spain. The productive cycle of the Iberian pig is orientated towards a final grazing/fattening period in the *dehesa*, consuming acorns (1.3–6 kg dry matter (DM)/d; Rodríguez-Estévez et al., 2009) from oak-trees (*Quercus ilex rotundifolia* and *Quercus suber*) as the only feed source, complemented with pasture when available and provide products of exceptional organoleptical properties (López-Bote, 1998). Acorn feeding is a radical dietary shift for pigs following intensive rearing with nutritionally balanced diets. Although the acorn is very palatable to pigs, the protein content (48–63 g/kg DM) is very low and the amino acid (AA) profile unbalanced, where lysine is the first limiting AA (Nieto et al., 2002a); moreover, it is rich in starch and lipids (815–843 and 77–121 g/kg DM, respectively). Recommendations derived from modern breeds have been used in Iberian pigs, even though there are evidences that they have distinct metabolic (Fernández-Fígares et al., 2007) and nutritional features (Nieto et al., 2012). Although ileal digestibility of AA of acorn protein has been measured in Iberian pigs (Nieto et al., 2002a; García-Valverde et al., 2007), no information is available on their net portal appearance (NPA). In a recent manuscript (Fernández-Fígares et al., 2018a), we have evaluated the NPA of metabolites of Iberian pigs fed acorns and we found out that ammonia decreased after one week of acorn consumption. It is important to know if decreased NPA of ammonia was a consequence of a reduction in AA metabolism at the PDV level which would result in increased NPA of AA. We hypothesized that adaptation of pigs eating equilibrated diets to acorn feeding increases NPA of AA. The aim of the present study was to determine NPA of AA in Iberian pigs fed with acorn from evergreen oak (*Quercus ilex rotundifolia*) and if there was an adaptation effect to a very low protein diet. The information could be used to establish recommendations of AA supplementation when Iberian pigs are in grazing conditions.

## Methods

### Animals, facilities, and diet analysis and composition

The study protocol was approved by the Bioethical Committee of the Spanish Council for Scientific Research (CSIC, Spain; project reference RECUPERA 2020, FEDER funding).

Six Iberian (Silvela strain; Sánchez Romero Carvajal, Jabugo S.A., Puerto de Santa María, Cádiz, Spain) gilts of similar BW (25 ± 0.4 kg initial body weight (BW)) were utilized. One week before surgery, pigs were housed in individual pens (2 m^2^) in a controlled environment room (21 ± 1.5 °C), whit *ad libitum* access to a standard diet (145 g crude protein (CP)/kg DM and 14.3 MJ metabolizable energy (ME)/kg DM) based on barley-soybean meal and free access to water. After surgery, pigs were on metabolic cages and fed the standard diet until the previous day to blood sampling at 2.5 × ME for maintenance (422 kJ/kg^0.75^ BW/d; Nieto et al., 2002b) in two portions, at 09:00 (0.25) and at 15:00 h (0.75). The day before the first sampling, the standard diet was substituted for a non-supplemented acorn diet following the feeding protocol described above. Acorn diet consisted of 2.4 kg of acorn providing 1.425 kg DM and a total CP intake of 74.1 g and continued until the final of sampling periods.

**Table 1:** Nutritional composition of the kernel of acorns used in the study (g/kg dry matter).

Dry matter	735
Ash	18
Organic matter	982
Fat (ether extract)	65.0
Nitrogen	8.3
Crude protein	52.0
aNDFom^a^	41.8
ADFom^b^	16.7
Lignin(sa)^c^	2.6
Amino acids	
Alanine	2.28
Arginine	5.26
Aspartic acid	6.89
Cysteine	0.70
Glutamic acid	5.78
Glycine	2.13
Histidine	1.58
Isoleucine	1.80
Leucine	3.49
Lysine	2.63
Methionine	0.79
Phenylalanine	1.95
Proline	2.84
Serine	1.81
Threonine	1.80
Tyrosine	1.38
Valine	2.37
EAA^d^	21.7
NEAA^d^	23.8
Total AA^d^	45.5
Gross energy (MJ/kg DM)	15.1


**Notes.**

a Neutral detergent fiber assayed with a heat stable amylase and expressed exclusive of residual ash.

b Acid detergent fiber expressed exclusive of residual ash.

c Lignin determined by solubilization of cellulose with sulphuric acid.

d Sum of essential (EAA), non-essential (NEAA) and total amino acids (AA).

Iberian pigs in extensive production do not eat the whole fruit acorn but the kernel, discarding the hull. Composition and chemical analysis of the diet was performed by standard procedures (AOAC, 2000) and can be found elsewhere (González-Valero et al., 2016a). The nutrients composition of acorn kernels fed is shown in Table 1. DM (no. 934.01), ether extract (no. 920.39) and total ash (no. 942.05) analysis were performed by standard procedures (AOAC, 2000). Total N was determined according to the Dumas’ method, by total combustion in TruSpec CN equipment (Leco Corporation, St. Joseph, MI). CP was determined as total N × 6.25. The neutral, acid and lignin detergent fractions (aNDFom (NDF assayed with a heat stable amylase and expressed exclusive of residual ash), ADFom (ADF expressed exclusive of residual ash) and Lignin(sa) (lignin determined by solubilization of cellulose with sulphuric acid), respectively) in kernels were analyzed by the method of Goering & Van Soest (1970). Neutral and acid detergent fiber was determined using an ANKOM^220^ Fibre Analyser Unit (ANKOM Technology Corporation, Macedon, NY, USA). Gross energy was measured in an isoperibolic bomb calorimeter (Parr Instrument Co., Moline, IL). AA composition of acorn kernel was determined after protein hydrolysis in 6 mol/L hydrochloric acid plus 10 g/kg phenol in sealed tubes at 110 °C for 24 h, by high performance liquid chromatography (HPLC) using the Waters Pico-Tag method for hydrolysates. It involves pre-column derivatization with phenylisothiocyanate (Cohen, Meys & Tarvin, 1989) and a Waters Nova-Pak C18 phase reverse column (4 µm, 3.9 × 150 mm). Cysteine and methionine were determined as cysteic acid and methionine sulphone, respectively, obtained by oxidation with performic acid before protein hydrolysis (Moore, 1963). Tryptophan was not determined. Free AA in plasma were determined by HPLC using the Waters Pico-Tag method for physiological AA using a reverse phase column (Waters Pico-Tag, 3.9 × 300 mm) and pre-column derivatization with phenylisothiocyanate (Cohen, Meys & Tarvin, 1989).

### Experimental procedures, schedules, analysis and calculations

The day before surgery (28 kg average BW) pigs were fasted and water removed. Three chronic indwelling catheters were placed: in portal vein and carotid artery for blood sampling, and in ileal vein for para-aminohippuric acid (PAH; 2% w/v; Sigma-Aldrich Química S.A., Madrid, Spain) infusion as a marker to measure blood flow. Detailed description of the catheters design, construction and maintenance, surgical procedure and post-surgery care of pigs was described previously (Rodríguez-López et al., 2013).

Pigs were adapted to close contact with the staff involved in sampling to reduce stress to a minimum. Two sampling periods were carried out under identical conditions when pigs were completely recovered from surgery. Then pigs were changed from the standard diet to a non-supplemented acorn diet (34 kg average BW) following the feeding protocol described above. First sampling (sampling period 1) was carried out one day after the diet change and second sampling (sampling period 2) was made seven days later.

The day of sampling, an initial 300 mg pulse dose of PAH was administered into ileal vein 45 min prior blood sampling (Yen & Killefer, 1987), followed by a continuous infusion of 16 mg/min using a syringe pump (Model 33, Harvard Apparatus Inc., Holliston, MA, USA). Apyrogenic filters (MILLEX GP, Syringe Driven Filters Unit, 0.22 µm; Millipore, Carringtwohill, Ireland) fitted infusion syringes. Blood samples using 4.5 mL heparinized tubes (Monovette VetMed, Sarstedt, Nümbrecht, Germany) were taken simultaneously from carotid artery and portal vein at -5 min, 0.5, 1, 1.5, 2, 2.5, 3, 3.5, 4, 5 and 6 h after feeding 0.25 of total daily acorn ration. Haematocrit was determined using a microcentrifuge (11,500 × g for 5 min; Biocen, Orto-Alresa, Ajalvir, Madrid, Spain). Plasma was obtained by centrifugation (4 °C and 1820 × g for 30 min) and stored at −20 °C until PAH (Lobley et al., 1995; Fernández-Fígares et al., 2018b) and AA analyses. Because of co-elution with ammonia, threonine was not quantified in plasma. After sampling, pigs were fed the remaining of the daily acorn allowance. Pigs continued having 2.4 kg of acorn in the same proportions (at 09:00 (0.25) and 13:00 h (the remaining 0.75)) for one week after which the second sampling period (period 2) was carried out following identical protocol. Portal blood flow (PBF) and portal plasma flow (PPF) were determined by the indicator dilution method using haematocrit and plasma PAH concentrations (Katz & Bergman, 1969). The PBF and NPA of AA were calculated according to the Fick principle of arteriovenous concentration difference and flow rate (Zierler, 1961). The NPA of AA was calculated by multiplying the porto-arterial plasma concentration difference of AA by PPF. Dietary AA intake was calculated to determine the fractional absorption for each AA (ratio of AA appearing in the portal vein during the 6 h postprandial period, relative to the intake).

### Statistical analyses

The experimental unit was the pig and measurements were made sequentially over time in each pig. Data were subjected to repeated-measures analyses, using the mixed model procedure of SAS (SAS Institute, Cary, NC, USA). The main effects in the model were the sampling period (1 and 2), time (0.5, 1, 1.5, 2, 2.5, 3, 3.5, 4, 5 and 6 h) and the interaction. The Kolmogorov–Smirnov test was used to ensure that data were normally distributed. Homogeneity of variances was assayed with Bartlett’s test. The differences were considered significant when *P* < 0.05 and a trend when 0.05 < *P* < 0.10.

## Results

Average PPF was 607 and 841 mL/min in sampling periods 1 and 2, respectively. It peaked at 0.5 and 1 h for periods 1 and 2, respectively, and decreased to basal rate thereafter. We observed no differences in the time needed to consume the test ration in both sampling periods.

**Figure 1:**
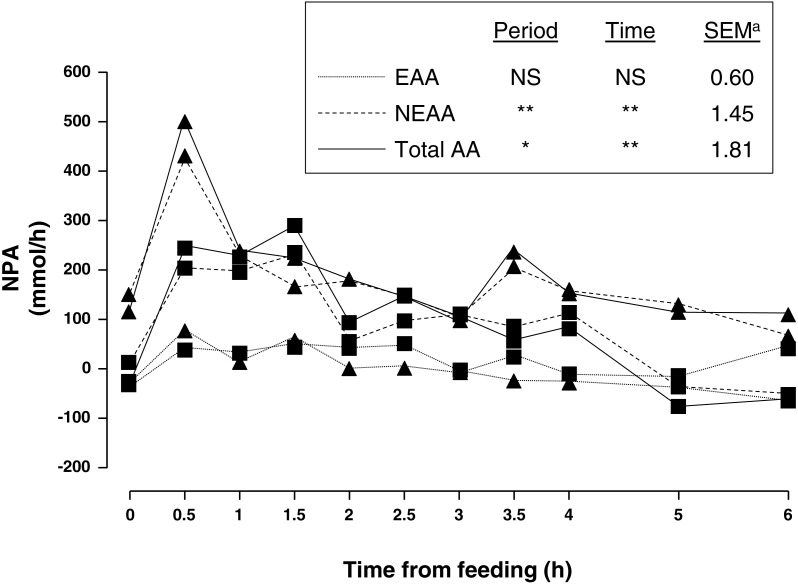
Net portal appearance (NPA) of essential (EAA), non-essential (NEAA) and total amino acids (AA) along a 6 h sampling in pigs (*n* = 6) fed acorns for 1 and 8 days (period 1 (■) and 2 (▴), respectively). a, Standard error of mean; ^∗^*P* < 0.05, ^∗∗^*P* < 0.01, NS, not significant (*P* > 0.05).

Average portal and arterial concentration, and NPA (Fig. 1) of AA during the 6 h sampling are shown in Table 2. There was no sampling period × time interaction for any of the AA.

**Table 2:** Portal and arterial content of essential (EAA), non-essential (NEAA) and total amino acids (AA), and net portal appearance (NPA) of AA in pigs (*n* = 6) fed acorns in sampling period (P) 1 and 2.

				*P*-value		
	P-1	P-2	SEM^b^	Period (P)	Time (T)	P × T
Portal AA (µmol/L)						
EAA	916	919	23.7	0.9427	0.0079	0.9727
Arginine	61.0	54.5	4.65	0.3270	0.0191	0.2664
Histidine	37.1	36.5	1.72	0.8210	0.5400	0.9512
Isoleucine	118	130	2.2	0.0005	<0.0001	0.8817
Leucine	110	126	5.9	0.0649	0.6263	0.9997
Lysine	153	162	6.6	0.3631	0.0155	0.9367
Methionine	16.0	12.2	1.15	0.0220	0.3927	0.9849
Phenylalanine	66.1	62.5	1.47	0.0864	0.0001	0.9264
Tryptophan	31.6	29.9	1.14	0.0005	0.6256	0.9315
Valine	303	309	5.9	0.4299	0.0027	0.9809
NEAA	2,259	2,412	44.5	0.0173	<0.0001	0.7240
Alanine	347	402	11.7	0.0016	<0.0001	0.9894
Asparagine	128	145	5.3	0.0310	<0.0001	0.5277
Aspartic acid	21.1	15.8	1.15	0.0021	0.0001	0.9169
Citrulline	34.9	26.2	1.56	0.0002	0.9041	0.9979
Cysteine	130	77.1	5.55	<0.0001	0.9685	0.8916
Glutamic acid	385	316	16.0	0.0032	0.9778	0.9802
Glutamine	37.8	63.6	4.68	0.0002	0.9360	0.8465
Glycine	526	666	15.4	<0.0001	0.9870	0.9532
Hydroxyproline	36.6	41.3	1.29	0.0126	0.9985	0.9769
Ornithine	130	117	5.2	0.0739	0.1716	0.9611
Proline	176	242	7.6	<0.0001	0.0001	0.9975
Serine	177	161	4.4	0.0175	0.0505	0.7456
Taurine	53.4	52.1	3.46	0.7880	0.7506	0.9916
Tyrosine	61.1	58.3	2.26	0.3811	0.0224	0.9512
Total AA	3,175	3,331	59.8	0.0692	<0.0001	0.7557
Arterial AA (µmol/L)						
EAA	918	898	25.4	0.5745	0.0617	0.9878
Arginine	64.4	70.6	3.84	0.2537	0.0001	0.9340
Histidine	35.7	31.4	1.71	0.0784	0.2961	0.9998
Isoleucine	121	133	2.7	0.0024	0.0028	0.9578
Leucine	104	117	5.2	0.0834	0.5661	0.9992
Lysine	148	151	7.3	0.8015	0.1039	0.9968
Methionine	15.2	12.2	1.02	0.0400	0.6656	0.9961
Phenylalanine	61.8	56.4	1.49	0.0123	0.0592	0.9821
Tryptophan	33.6	27.9	1.87	<0.0001	0.9471	0.9970
Valine	303	301	5.7	0.7692	0.0805	0.9629
NEAA	2,063	2,141	43.3	0.2053	0.0029	0.8941
Alanine	296	316	9.6	0.1380	<0.0001	0.9497
Asparagine	112	120	4.8	0.2251	<0.0001	0.6636
Aspartic acid	16.7	14.3	1.09	0.1224	0.0144	0.9782
Citrulline	27.8	19.5	1.17	<0.0001	0.9884	0.9999
Cysteine	124	75.7	3.78	<0.0001	0.4663	0.9704
Glutamic acid	425	341	17.1	0.0009	0.7680	0.9629
Glutamine	53.5	71.9	5.4	0.0194	0.9974	0.9695
Glycine	460	599	14.9	<0.0001	0.8897	1.0000
Hydroxyproline	33.6	36.5	1.18	0.0907	0.8608	1.0000
Ornithine	92.5	79.8	3.13	0.0056	0.0100	0.9875
Proline	145	197	5.9	<0.0001	0.0031	0.8179
Serine	172	156	4.4	0.0124	0.3768	0.8671
Taurine	46.1	44.5	2.78	0.6759	0.8184	1.0000
Tyrosine	58.0	52.9	2.30	0.1246	0.4188	0.9838
Total AA	2,972	3,039	60.2	0.4350	0.0062	0.8815
NPA (µmol/min)						
EAA	3.27	16.8	10.01	0.3422	0.0820	0.6184
Arginine	−9.67	−13.1	3.858	0.5235	0.1490	0.4716
Histidine	1.61	4.66	0.542	0.0002	0.5984	0.6401
Isoleucine	−1.08	−1.48	1.174	0.8250	0.0097	0.9470
Leucine	3.91	6.86	1.064	0.0560	0.0019	0.7667
Lysine	3.76	8.72	2.477	0.1608	0.2340	0.7211
Methionine	−0.28	−0.36	0.570	0.9247	0.7405	0.9011
Phenylalanine	3.03	5.00	0.712	0.0544	0.0006	0.4212
Tryptophan	−1.74	−0.95	0.853	0.5129	0.2677	0.6125
Valine	−0.77	6.42	2.789	0.0726	0.5301	0.6007
NEAA	128	224	24.2	0.0064	0.0079	0.8137
Alanine	34.8	75.7	6.31	<0.0001	<0.0001	0.9997
Asparagine	10.1	20.6	3.28	0.0275	0.0076	0.1271
Aspartic acid	2.07	1.35	0.845	0.5471	0.3153	0.9939
Citrulline	4.41	5.49	0.556	0.1757	0.9257	0.6966
Cysteine	3.59	0.91	2.964	0.5244	0.4830	0.8121
Glutamic acid	−17.9	−21.0	7.78	0.7781	0.7730	0.8877
Glutamine	−9.05	−0.16	3.574	0.0852	0.9498	0.6963
Glycine	43.1	63.3	7.49	0.0643	0.1866	0.4068
Hydroxyproline	1.82	4.07	0.568	0.0066	0.4846	0.6098
Ornithine	20.8	30.3	2.97	0.0278	0.0407	0.6974
Proline	21.4	35.2	2.88	0.0011	<0.0001	0.7249
Serine	1.43	3.74	2.39	0.4967	0.9048	0.5724
Taurine	4.02	5.34	0.908	0.3100	0.7747	0.3716
Tyrosine	2.21	4.41	0.776	0.0503	0.0033	0.2425
Total AA	135	241	30.2	0.0156	0.0077	0.8606

**Notes.**

a Values are mean for ten postprandial measurements (0.5, 1, 1.5, 2, 2.5, 3, 3.5, 4, 5 and 6 h after feeding).

b Standard error of mean.

Amongst essential AA (EAA), valine had the greatest portal and arterial concentration and lysine was second. As for non-essential AA (NEAA), glycine had the greatest portal and arterial concentration followed by alanine and glutamic acid. As expected, feeding increased concentration of portal and arterial AA as well as NPA. There was a peak at 0.5 h after feeding and a gentle decrease thereafter. In general, portal was greater than arterial concentration of sum of NEAA and total AA as indicated by positive NPA at all times. Nevertheless, NPA of sum of EAA was positive from 0.5–2.5 h sampling and negative from 3–6 h sampling (*P* = 0.08) indicating an increased use of AA by the PDV after 3 h of acorn intake.

Adaptation to one week of acorn feeding (period effect) had no effect on portal and arterial concentration, and NPA (*P* > 0.10) of sum of EAA although some individual AA were affected. Nevertheless, portal concentration and NPA (*P* < 0.05) of sum of NEAA were affected after one week of acorn consumption. NPA of sum of NEAA and total AA increased (75 and 79%, respectively; *P* < 0.05) in period 2. Thus, NPA of NEAA alanine, asparagine, glutamine, glycine, hydroxyproline, ornithine, proline and tyrosine was 118, 104, 98, 47, 124, 46, 64 and 100%, respectively, greater (0.001 < *P* < 0.09) in sampling period 2. Similarly NPA of EAA histidine, leucine, phenylalanine and valine increased (189, 75, 65 and 934%, respectively; 0.001 < *P* < 0.08) after adaptation to acorn consumption. NPA was negative for glutamic acid and arginine and to a lesser extent for glutamine, isoleucine, methionine, tryptophan and valine (only period 1). NPA of NEAA quantitatively represented the major part of NPA of total AA (94% on average).

Fractional absorption of dietary EAA, NEAA and total AA is displayed in Table 3. It was negative for arginine, isoleucine, methionine and valine (period 1) among EAA, and glutamic acid. Fractional absorption of sum of EAA, NEAA and total AA was 97, 44 and 49% lower in sampling period 1 compared to 2.

**Table 3:** Fractional absorption (net portal appearance (NPA)/amino acids (AA) intake; mmol) of essential (EAA), non-essential (NEAA) and total AA of pigs (*n* = 6) fed acorns during a 6 h sampling in periods (P) 1 and 2.

	NPA		AA intake	Fractional absorption	
	P-1	P-2		P-1	P-2
EAA^a^	0.18	6.02	39.2	0.005	0.154
Arginine	−3.48	−4.72	8.70	−0.400	−0.542
Histidine	0.58	1.68	2.92	0.198	0.574
Isoleucine	−0.39	−0.53	3.94	−0.099	−0.135
Leucine	1.41	2.47	7.65	0.184	0.323
Lysine	1.35	3.14	5.19	0.261	0.605
Methionine	−0.10	−0.13	1.53	−0.066	−0.085
Phenylalanine	1.09	1.80	3.40	0.321	0.529
Tryptophan	−0.63	−0.34	–	–	–
Valine	−0.28	2.31	5.82	−0.048	0.397
NEAA^a^	32.6	58.7	57.6	0.566	1.019
Alanine	12.5	27.3	7.37	1.699	3.696
Asparagine	3.64	7.42	–	–	–
Aspartic acid	0.75	0.49	14.9	0.050	0.033
Citrulline	1.59	1.98	–	–	–
Cysteine	1.29	0.33	1.66	0.778	0.197
Glutamic acid	−6.44	−7.56	11.3	−0.570	−0.669
Glutamine	−3.26	−0.06	–	–	–
Glycine	15.5	22.8	8.15	1.904	2.796
Hydroxyproline	0.66	1.47	–	–	–
Ornithine	7.49	10.9	–	–	–
Proline	7.70	12.7	7.10	1.085	1.785
Serine	0.51	1.35	4.97	0.104	0.271
Taurine	1.45	1.92	–	–	–
Tyrosine	0.80	1.59	2.19	0.363	0.724
Total AA^a^	32.8	64.7	96.8	0.339	0.668

**Notes.**

a Sum stablished only for analyzed dietary AA.

## Discussion

In this study the animals were fed an amount of feed (0.25) proportional to the measurement period (6 h), simulating as much as possible that Iberian pigs spend the whole day grazing to cover their nutritional requirements (Rodríguez-Estévez et al., 2009). It is important to note that under real production situations herbage, when available, may represent a meaningful feed resource in grazing pigs when acorn is scarce. Nevertheless, under typical circumstances acorn consumption represents most of daily intake in grazing pigs (0.88 of dry matter as average; Rodríguez-Estévez et al., 2009) and that is why we have focused on acorn consumption. Iberian pigs in extensive production do not eat the whole fruit acorn but the inner kernels (average tannin content of 10 g/kg DM). The content of acorn tannins in the kernel is approximately 4 times minor than in the hull (Nieto et al., 2002a) consuming much less tannins than the total acorn content. There is supporting evidence (García-Valverde et al., 2007) that in this context tannins content is unlikely to influence digestion.

Iberian pigs are 92–115 kg BW at the beginning of the fattening phase in the Mediterranean forest and are slaughtered at 150 kg BW on average. Because surgery on heavy animals is cumbersome and expensive compared to younger pigs, we used 25 kg BW pigs for the study. Although the use of young pings as an appropriate model for mature pigs could be questioned, protein digestibility and balance studies performed in our Department (Nieto et al., 2002a; García-Valverde et al., 2007) support the use of the growing Iberian pig as model for heavy Iberian pigs. However, NPA values may be affected by the fact that nutrient requirements in young and adult animals do differ.

Overall, NPA of AA in the present study reached a maximum (30 min postprandial) and decreased gradually, in agreement with the literature (Lenis et al., 1996; Van der Meulen et al., 1997; Agyekum et al., 2016). However, time to reach the peak was shorter than in other studies as a consequence of the low amount of acorns offered as there is an inverse relationship between amount of nutrients intake (i.e., meal size) and time needed to absorb them at PDV level. It may be considered that under free range conditions Iberian pigs eat along the whole day having a more or less stable intake as compared with intensive feeding when pigs are fed once or twice a day. PBF measured in our conditions was within the range of measurements in Landrace and Iberian pigs fed an amount of feed proportional to the measurement period (970–1,357 mL/min (Rodríguez-López et al., 2010); 746–1,133 mL/min (González-Valero et al., 2016a)). Differences in PBF observed in the literature could be due to different experimental conditions. Although noticeable, hyperemia in the present study was probably of small magnitude due to the small amount of feed (<15 g diet/kg BW/d and 6.5 g of protein/kg BW/d) offered before sampling (Fara, 1984) and/or to the low protein content of acorns. Indeed a relation between feed intake and blood flow has been proved (Lomax & Baird, 1983; Huntington, 1984). The increased PPF in sampling period 2 may indicate a change in PDV physiology induced by an adaption to acorn diet. Acorn protein content is considerably low (52 g/kg DM), less than 0.3 times that of a standard diet for Iberian pigs.

Concentrations of AA in the portal vein and carotid artery were similar to those we previously reported for Iberian pigs (González-Valero et al., 2012) fed isoenergetic barley–soybean meal diets of low and high CP content (130 and 160 g/kg DM, respectively). As expected, feeding increased concentrations of portal and arterial AA, although the increase in the arterial level observed after the meal is less marked in the peripheral than in the portal circulation because of the uptake of AA by the liver. The similar arterial concentrations of total AA in both sampling periods could be interpreted as comparable net retention of protein inasmuch as arterial AA represent a composite pool integrating absorption and the balance between protein synthesis and breakdown.

It is difficult to compare results of NPA of AA from different studies as NPA is affected by numerous factors (protein content, fibre, ME intake, etc.; Rérat, 1993). Greater NPA of AA have been reported in pigs fed diets of higher CP content (120–240 g/kg DM) than in the current study (from 1,371–2,764 µmol/min for total AA (Large White pigs; Simoes Nunes et al., 1991); 18 and 3.6 µmol/min for lysine and methionine, respectively (Iberian pigs; González-Valero et al., 2012)). This can also be ascribed to a greater gut endogenous protein secretion and AA reabsorption after digestion in pigs fed diets with increasing protein content (Nyachoti et al., 1997). Fractional absorption of AA needs to be carefully interpreted being aware of the limitations of the technique as for example the influence of endogenous losses of protein. Fractional absorptions of most AA in the current study were of lower magnitude that than corresponding acorn AA digestibility values in the literature (Nieto et al., 2002a) although it is difficult to know when all AA have been digested. However, postprandial NPA of AA reached the preprandial level (Fig. 1) after six hours, indicating that it was enough time for the AA to be absorbed under our experimental conditions. Lysine and sum of total AA had comparable digestibility and fractional absorption values. Although lysine is the first limiting AA in acorns according to chemical score, NPA and fractional absorption were high compared to other EAA. Similarly, lysine was the first limiting AA in milk-protein fed to piglets according to chemical score (Davis et al., 1994) while threonine and methionine were the limiting AA according to net portal AA balance (Stoll et al., 1998). Amongst EAA, arginine showed the lowest NPA and fractional absorption so arginine could be considered the first limiting AA followed by tryptophan (only referred to NPA), isoleucine and methionine in Iberian pigs fed acorns. Dietary arginine is highly metabolized during intestinal transport to provide ornithine and citrulline in pigs (Yin et al., 2010; Wu et al., 2016), but unlike glutamine, arginine is not oxidized at the small intestine (Blachier et al., 1991). Fractional absorption and NPA of valine (only period 1), arginine, isoleucine, methionine and glutamic acid were negative, that is, the net use of these AA by the PDV was greater than the dietary intake, implying a high rate of metabolism in gastrointestinal tissues.

Although NPA of histidine, leucine, phenylalanine and valine increased (*P* < 0.1) in period 2, the corresponding fractional absorption was still very low. Fractional absorptions of EAA between 0.5–1 have been reported using diets with greater protein content (Stoll & Burrin, 2006).

Pigs in our experiment consumed a protein deficient diet that could probably alter intestinal epithelia and maintaining the proper function and health of the intestinal epithelium under this situation would increase the requirement for all AA -especially arginine and histidine-, in line with a low NPA of these AA. Reduced height of the intestinal villi was found in pigs fed a nutrient deficient diet (Morales et al., 2016).

Leucine fractional absorption reported herein is much lower than values in pigs (0.40–0.55 (Yin et al., 2010); 0.50–0.68 (Van der Schoor et al., 2001)) fed diet with greater CP content. There is significant leucine metabolism by the gut via both transamination and complete oxidation to CO_2_ (Stoll & Burrin, 2006). Studies in grower pigs suggest that approximately 0.40 of the whole-body phenylalanine oxidation occurs in the PDV tissues (Bush et al., 2003), in line with our results.

Glutamic acid, glutamine and, to a lesser extent, aspartic acid appear to be significant oxidative fuels in the intestine, as reflected by their low or even negative NPA. Despite the importance of glucose as oxidative fuel, Stoll et al. (1999) obtained that the proportion of glucose oxidized completely to CO_2_ was substantially less than that of either glutamine or glutamate in piglets, in agreement with the positive NPA of glucose with the same animals of the present experiment (Fernández-Fígares et al., 2018a). A nearly complete first-pass removal of dietary glutamate and aspartate has been reported in pigs (Stoll et al., 1998).

On the other hand, NPA of alanine, glycine and proline were the largest in the conditions of our experiments and their fractional absorption was above 1. This means that there was a net synthesis at the PDV level. High NPA of alanine and glycine are the result of metabolic processes in the gut wall (Lenis et al., 1996; Stoll et al., 1998). Glutamine and glutamate act as precursors for proline synthesis (Watford, 2008), which is increased during intestinal wound repair (Morales et al., 2016). It follows that the lower NPA of glutamine (*P* < 0.09) and proline (*P* < 0.01) in sampling period 1 may represent an increased requirement, as a result of mucosal damage elicited by the low protein diet. Nevertheless, it is worthy to mention that NPA of AA is affected by endogenous proteins, as proline rich mucins, which were not determined in our study and which are expected to have an important contribution in low protein diets. Heat stress and reduced feed intake decrease intestinal integrity in pigs (Pearce et al., 2013). In post-absorptive state, the intestine releases large amounts of citrulline together with alanine and proline in pigs (Wu, Borbolla & Knabe, 1994) indicating *de novo* synthesis by the gut. However, NPA of citrulline, immediate precursor of arginine, was relatively low in our study decreasing the pool of available citrulline for arginine synthesis. Indeed, dietary citrulline supplementation was more efficient to increase arginine availability than arginine supplementation in mice (Marini, Agarwal & Didelija, 2016). NPA of citrulline of 27 kg BW Yorkshire × Landrace × Duroc pigs fed at 2.8 × ME for maintenance and 8.2% CP diet deficient in NEEA but not in EAA was 10.85 µmol/min (Mansilla et al., 2018), which is considerably higher than the values obtained herein. Ornithine originates from the metabolism of dietary and blood arginine (Windmueller & Spaeth, 1980), in agreement with the large ornithine concentration in portal blood and positive NPA in this and other studies (Rérat, 1993). The absorption of dietary cysteine into portal blood is very limited in young pigs (less than 0.20 of dietary intake), implying extensive intestinal use of cysteine in first-pass (Stoll et al., 1998; Bos et al., 2003), as a precursor for glutathione synthesis. In our conditions, fractional absorption was close to this value in period 2 probably indicating increased glutathione requirement compared to period 1.

Overall, we found that AA are considerably used by PDV when Iberian pigs are fed acorn as indicated by their reduced fractional absorption, although adaptation to acorn elicited a significant improvement in AA availability for the liver and peripheral tissues. In our study, fractional absorption in pigs adapted to acorn feeding (0.668) are comparable to literature values (0.64 (Stoll et al., 1998) or 0.69 (Yin et al., 2010)). NPA of ammonia with the same animals of the present study (Fernández-Fígares et al., 2018a) decreased in sampling period 2 in parallel with increased NPA of EAA, NEAA and total AA indicating an overall lower catabolism of AA by the PDV so that more AA are available for productive tissues although some carry over effect of the standard diet cannot be completely discarded. This is in accordance with lower plasma urea (Fernández-Fígares et al., 2018a) and consequently reduced N excretion in the urine after one week of acorn feeding. Although bacteria in the lumen of the pig small intestine may utilize nutritionally EAA and NEAA for both oxidation and protein synthesis (Dai et al., 2010), the low amount of protein ingested is at odds with a significant bacterial fermentation in the gut. Additionally, no differences were found in NPA of urea (Fernández-Fígares et al., 2018a) and volatile fatty acids (González-Valero et al., 2016b) between periods in our experiments, which is in line with similar bacterial fermentation in the gut. It has been reported that visceral AA oxidation was substantially suppressed in low (0.4 g protein/kg BW/h) compared to high protein diets (0.9 g protein/kg BW/h; Van der Schoor et al., 2001). It could be speculated that AA oxidation was low in favor of increased synthesis of protein and of other AA and derived molecules at the PDV level in pigs fed a very low amount of protein (0.11 g protein/kg BW/h; present study).

The biological explanation for the differences in the proportion of dietary AA utilized by the gut is only partially understood and requires further study to specifically establish the functional purpose of each AA.

## Conclusions

The NPA of AA of Iberian pigs fed acorns was very low. After one week of acorn feeding, pigs underwent an adaptation increasing AA available for peripheral tissues. Strategies aimed at supplementing key gut-nutrients in support of gut function may improve growth, so supplementation with certain AA during the grazing period in Iberian pigs is recommended. The study of NPA of AA after herbage intake would add a more profound insight on protein metabolism and warrants further research.

##  Supplemental Information

10.7717/peerj.6137/supp-1 Supplemental Information 1 Raw data used to calculate values displayed in the article

## Additional Information and Declarations

### Data Availability

The following information was supplied regarding data availability: The raw data are provided in a Supplemental File.
